# Tourniquet‐Induced Atypical Lower Back Pain Under Spinal Anesthesia: A Case Report and Mechanistic Exploration of Referred Pain

**DOI:** 10.1155/carm/5531848

**Published:** 2026-06-29

**Authors:** Zhifu Zhao, Zhongjun Liu, Yakang Wang, Candida

**Affiliations:** ^1^ Department of Anesthesia, Deyang People’s Hospital, Deyang, China; ^2^ Department of Anesthesia, Meishan People’s Hospital, Meishan, China; ^3^ Department of Orthopedics, Ya’an People’s Hospital, Yaan, China, yasyy.com; ^4^ Department of Anesthesia, Central Hospital of Maputo, Maputo, Mozambique

**Keywords:** complications related to tourniquets, fractures, low back pain, spinal anesthesia

## Abstract

Tourniquet‐associated complications typically manifest as localized limb symptoms or systemic reactions. Here, we report a rare case of acute, severe lower back pain directly induced by a tourniquet during lower limb surgery under spinal anesthesia. A 32‐year‐old male developed intense lower back pain (VAS: 4–10) approximately 1.5 h after tourniquet application to the right thigh, accompanied by elevated blood pressure and agitation, while bilateral lower limb sensorimotor blockade remained intact (T11‐S5 level). Intravenous analgesics provided only transient and limited relief. Crucially, within approximately two minutes of pausing the procedure and releasing the tourniquet, the patient’s pain completely resolved (VAS: 0) without additional analgesia, and the anesthetic level was unchanged. This case provides the first clear documentation of tourniquet pain presenting as isolated truncal pain entirely remote from the ischemic region. We propose that this phenomenon arises because spinal anesthesia blocks somatic sensory input from the lower limbs, leading to nociceptive signals from ischemic muscle being transmitted via incompletely blocked visceral or sympathetic afferent pathways to the spinal cord. According to the convergence‐projection theory, these signals are then misinterpreted by the brain as originating from the lower back (T10‐L2 dermatomes). This finding reveals how regional anesthesia can fundamentally alter the clinical presentation of tourniquet pain, addresses a critical gap in the current understanding of this complication, and alerts clinicians to include atypical referred pain in their differential diagnosis to prevent misdiagnosis and excessive medical intervention.

## 1. Introduction

The tourniquet is an indispensable instrument for controlling life‐threatening limb hemorrhage and establishing a bloodless surgical field. However, its application is accompanied by a spectrum of adverse effects, collectively termed tourniquet‐associated complications, which encompass local compressive injury, nerve palsy, ischemia‐reperfusion damage, and most commonly, tourniquet pain. The literature typically characterizes tourniquet pain as numbness, dull ache, burning sensation, or intolerable discomfort localized to the limb distal to the tourniquet or at the cuff site. Research has demonstrated that the incidence of tourniquet pain ranges from 51.8% to 60% in the upper extremities and from 7.7% to 35.8% in the lower extremities [1]. Notably, the occurrence of tourniquet pain correlates positively with the duration of application and occurs more frequently in patients receiving regional anesthesia [1, 2]. Female patients exhibit a higher incidence, earlier onset, and longer duration of tourniquet pain [3]. Furthermore, in comparison with pneumatic tourniquets, nonpneumatic elastic tourniquets (Esmarch bandages) are associated with a significantly higher incidence of tourniquet‐related pain and neurologic injury [4]. Prevailing pathophysiological explanations focus on localized tissue compression, ischemia, accumulation of metabolites, and cortical modulation [5].

A critical yet underexplored question is how nociceptive signals arising from tourniquet‐induced ischemia are expressed when the primary peripheral pain afferent pathways are pharmacologically blocked by regional anesthesia, such as spinal blockade. The prevailing assumption is that pain would either remain confined to the anesthetized region or manifest as generalized sympathetic excitation. Herein, we report an extreme case in which, under spinal anesthesia, tourniquet pain completely “translocated” to the nonanesthetized lower back. This phenomenon challenges conventional descriptions and reveals a significant cognitive blind spot regarding the expression patterns of tourniquet pain under altered neural conduction conditions. This report details the clinical case, explores its potential mechanisms within the framework of pain physiology, and aims to enhance clinical recognition while stimulating further investigation.

## 2. Case History/Examination

A 32‐year‐old male patient (height: 172 cm, weight: 71 kg) was admitted for a scheduled open reduction and internal fixation with plate and screws, following a right lateral tibial plateau fracture sustained in a traffic accident 14 days prior. The patient had an unremarkable past medical history, with no reported chronic conditions such as hypertension, cardiac disease, bronchial asthma, lumbar disc herniation, or neuropsychiatric disorders and no history of infectious diseases including hepatitis, tuberculosis, syphilis, or HIV. There was no prior surgical history or known drug allergies. Admission laboratory tests, including complete blood count and electrocardiogram, revealed no significant abnormalities. Imaging studies, comprising anteroposterior and lateral radiographs of the right lower limb, demonstrated a lateral tibial plateau fracture with mild lateral displacement, accompanied by knee joint effusion and a visible fat‐fluid level, indicative of lipohemarthrosis. Subsequent computed tomography (CT) confirmed a nondisplaced, nondepressed split fracture of the lateral tibial plateau, with joint effusion and a fat‐fluid level, consistent with the diagnosis of lipohemarthrosis. Physical examination revealed a conscious patient with no abnormalities in the head, trunk, or upper extremities. The spine exhibited normal physiological curvature without tenderness in the lumbar region. While the left lower limb demonstrated intact sensation and motor function, the right thigh showed normal contour and muscle strength without pain or deformity. Mild swelling and tenderness were noted over the proximal segment of the right tibia; however, sensation and motor function of the right toes were preserved. Examinations of the anus and external genitalia were unremarkable. The preoperative diagnosis was a right tibial plateau fracture (Schatzker Type I). Tibial plateau fractures are most commonly classified according to the Schatzker system, which stratifies injuries based on fracture location and degree of metaphyseal extension (Supporting Information 1). This widely recognized system defines six distinct fracture patterns as originally described by Schatzker et al. [6]. The right lower limb was immobilized in a plaster cast preoperatively until the start of surgery. No additional cast or orthosis was applied postoperatively.

**FIGURE 1:**
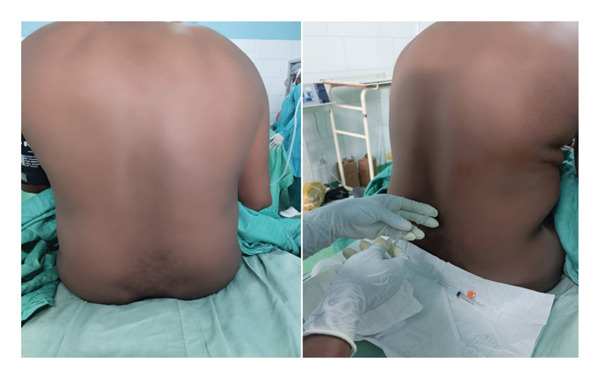
Spinal anesthesia puncture.

**FIGURE 2:**
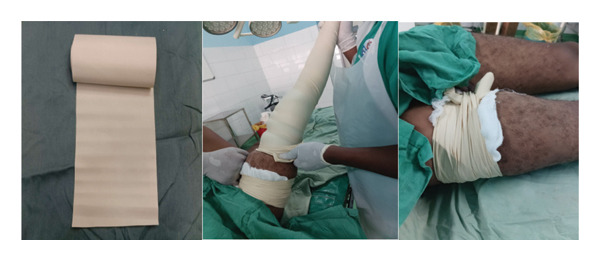
Tourniquet and binding tourniquet.

The patient, after a 10‐hour preoperative fast, was transferred to the operating room at 08:45. Upon arrival, he was conscious and calm and exhibited stable vital signs: pulse rate of 78 bpm, respiratory rate of 18 breaths/min, blood pressure of 131/84 mmHg, SpO_2_ of 99%, and temperature of 36.5°C. The anesthetic procedure commenced at 09:10 with the patient in a sitting position. Using the intercristal line (connecting the bilateral iliac crests) and the posterior midline as anatomical landmarks, the L3/4 intervertebral space was identified. Following standard surgical hand scrubbing, the area was prepared with three consecutive, overlapping applications of povidone‐iodine solution, adhering to aseptic technique from the center outward. A 25‐G fine spinal needle (AN‐S II, 103 mm, Zhejiang Runqiang Medical Instruments Co., No. 168 Dade Road, Xiuzhou District, Jiaxing City, Zhejiang, China) was then introduced into the subarachnoid space, guided by a 10‐mL syringe needle. The puncture was successful, with clear cerebrospinal fluid (CSF) observed upon dural penetration. A total of 4 mL of a mixed solution containing 100 μg (1 mL) of morphine and 15 mg (3 mL) of 0.5% bupivacaine was injected slowly through the needle (Figure 1). Completion of drug administration was noted at 09:18. The patient was immediately placed supine, and through meticulous adjustment of the operating table, the sensory blockade level was established and fixed between the T9 and S5 dermatomes. At 09:35, following exsanguination of the right lower limb from the foot to the proximal‐mid thigh by the orthopedic surgeon using a nonsterile, nonpneumatic elastic tourniquet (HD611/HD‐1580, Anji Hongde, estimated pressure value: 250–300 mmHg), a tourniquet was positioned at the proximal and middle third of the right thigh to achieve vascular occlusion of the right lower limb (Figure 2). The time of tourniquet inflation was documented, and a 1.5‐hour timer alarm was set.

The procedure commenced at 09:42 with the patient conscious and pain‐free. At 10:52, the patient reported lower back pain (visual analogue scale [VAS]: 4), accompanied by elevated vital signs; sensory blockade was assessed at T11‐S5. Analgesia was administered intravenously with 1 g of acetaminophen and 4 mg of morphine, supplemented by verbal reassurance. By 10:59, the patient described the pain as tolerable (VAS: 2), with a mild decrease in blood pressure and heart rate. Beginning at 11:06, pain intensity progressively increased, leading to a renewed rise in vital signs. By 11:13, the patient experienced severe lower back pain that was unresponsive to reassurance, manifesting as upper‐body lifting movements supported by both arms while the lower limbs remained paralyzed, with repeated reports of intolerable pain. Concurrently, the tourniquet time alarm activated. After consultation with the surgical team, the operation was paused at 11:15; the patient was instructed to breathe deeply while the tourniquet was gradually deflated, and preparations were made for additional potent analgesia. At 11:17, before any further analgesics were administered, the patient’s facial expression markedly improved, and he lay quietly, reporting complete pain resolution (VAS: 0). Surgery was promptly resumed and concluded at 12:15, with a total duration of 2 h and 33 min. Intraoperative fluid administration totaled 1300 mL, with an estimated blood loss of 150 mL and urine output of 400 mL. From 11:17 until the end of the procedure, the patient remained conscious, cooperative, and pain‐free, with a stable sensory blockade level maintained at T12‐S5.

**FIGURE 3:**
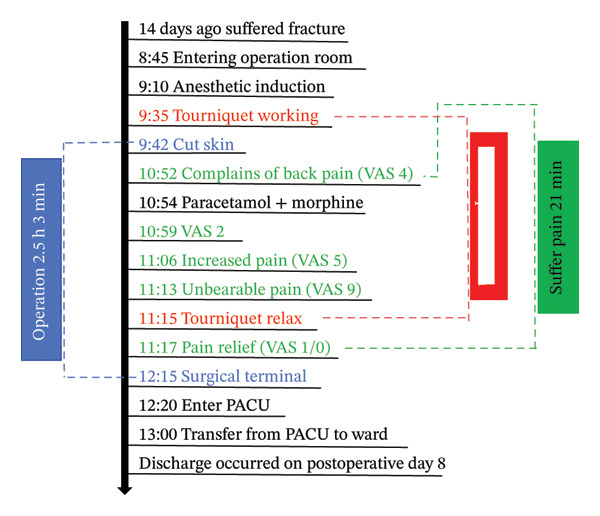
Timeline of episode.

Following intramuscular administration of 75 mg diclofenac sodium, the patient was transferred to the post‐anesthesia care unit (PACU) at 12:20. Vital signs remained stable throughout a 30‐minute continuous monitoring period (perioperative vital parameters are summarized in Table 1), and the patient was subsequently discharged to the orthopedic ward at 13:00. At 06:30 on the first postoperative day, the patient initially developed moderate‐to‐severe incisional pain at the right knee surgical site, with a VAS score of 5. Intramuscular injection of 100 mg tramadol was administered, which yielded substantial pain relief within 10 min and reduced the VAS score to 2. Recurrent incisional pain with a VAS score of 6 occurred at 14:00 on the same day, and repeated intramuscular tramadol (100 mg) rapidly alleviated symptomatic discomfort. A standardized multimodal analgesic regimen consisting of 1 g intravenous infusion of paracetamol combined with 75 mg intramuscular diclofenac sodium was initiated at 20:00 and administered at 6‐hour intervals for 72 consecutive hours. Following the implementation of this systematic analgesic strategy, no further pain episodes with a VAS score greater than 3 were observed throughout the subsequent postoperative course. The patient exhibited an uneventful postoperative recovery without additional adverse events or in‐hospital complications beyond intermittent surgical pain and was successfully discharged on the eighth postoperative day. The overall clinical timeline of this case is illustrated in Figure 3.

**TABLE 1:** Vital signs.

Time	BP	PR	RR	SpO_2_	T	Time	BP	PR	RR	SpO_2_	T	VAS
08:45	131/84	78	18	99	36.5	10:40	129/88	88	19	100	36.6	
08:50	124/76	75	18	99	36.5	10:45	132/89	89	20	100	36.6	
08:55	123/75	75	17	100	36.5	10:50	139/94	92	20	100	36.6	
09:00	122/74	73	18	100	36.5	10:55	144/97	96	20	100	36.6	4 (10:52)
09:05	124/75	73	18	100	36.5	11:00	131/94	90	21	100	36.7	2 (10:59)
09:10	119/64	81	19	99	36.5	11:05	138/99	97	22	100	36.7	
09:15	90/56	84	20	100	36.5	11:10	147/100	99	22	100	36.7	5 (11:06)
09:20	102/66	87	20	100	36.5	11:15	152/102	107	23	100	36.7	9 (11:13)
09:25	104/67	88	20	100	36.5	11:20	121/68	90	22	100	36.6	0 (11:17)
09:30	107/66	89	20	100	36.5	11:25	121/66	84	20	100	36.6	
09:35	109/65	84	20	100	36.5	11:30	122/74	83	20	100	36.6	
09:40	109/67	83	18	100	36.4	11:35	122/75	83	18	100	36.6	
09:45	109/63	83	18	100	36.4	11:40	123/74	83	19	100	36.5	
09:50	106/62	83	17	100	36.4	11:45	120/71	83	19	100	36.5	
09:55	105/60	82	17	100	36.4	11:50	121/73	83	19	100	36.5	
10:00	102/64	81	16	100	36.4	11:55	123/74	83	19	100	36.5	
10:05	106/66	81	16	100	36.4	12:00	124/77	84	19	100	36.4	
10:10	109/69	82	16	100	36.4	12:05	124/75	84	19	100	36.4	
10:15	116/72	83	16	100	36.5	12:10	123/74	83	19	99	36.4	
10:20	116/73	83	18	100	36.5	12:15	120/71	84	19	99	36.4	
10:25	120/74	83	18	100	36.5	12:20	122/76	84	19	99	36.5	
10:30	123/76	85	18	100	36.5	13:00	126/75	85	19	97	36.5	
10:35	127/84	88	19	100	36.5							

*Note:* BP = blood pressure (mmHg), PR = pulse rate (beats/min), RR = respiration rate (times/min), and T = temperature (°C).

## 3. Differential Diagnosis

Approximately 1.5 h after the establishment of effective anesthesia, the patient reported lower back pain. The anesthesiologist initially considered possible etiologies including regression of the anesthetic block, lumbar disc herniation, or discomfort from prolonged positioning on the operating table. Assessment revealed a sustained sensory blockade level of T11‐S5, ruling out complete anesthetic failure. Given the absence of a prior history of back pain, lumbar disc pathology was also deemed unlikely. Reassurance and intravenous acetaminophen were administered, yielding modest pain relief. As the definitive cause remained unclear, surgery proceeded with close monitoring, while the fact that tourniquet time had exceeded one hour was overlooked. As pain intensified, supplemental morphine provided only transient and suboptimal analgesia, and the patient became increasingly restless and less cooperative. The anesthesiologist maintained the presumption of block regression; however, the patient’s severe pain compromised accurate sensory level assessment, casting doubt on the previously documented T11‐S5 level. While preparations were being made to convert to general anesthesia, deflation of the tourniquet unexpectedly resulted in complete resolution of pain. The patient became calm and cooperative, and reassessment confirmed the sensory level remained at T11‐S5. This outcome established a direct causal relationship between tourniquet use and the onset of back pain, leading to a final diagnosis of a tourniquet‐associated complication.

## 4. Discussion

The causal link between tourniquet application and acute lower back pain in this case was definitively established through a clear “dechallenge” test, the immediate and complete resolution of pain upon tourniquet release. Intraoperatively, the pain was initially attributed to potential regression of the anesthetic level, positional discomfort, or an incidental lumbar issue. However, persistent confirmation of an intact sensory blockade, coupled with the dramatic symptom relief following tourniquet deflation, systematically excluded these alternative explanations. This underscores the critical importance of including tourniquet‐associated complications in the immediate differential diagnosis when unexplained truncal pain arises under regional anesthesia. Failure to consider this etiology risks misdiagnosis as “anesthetic failure,” potentially triggering unnecessary conversion to general anesthesia, excessive opioid administration, or procedural interruption, a clinical dilemma exemplified in the initial management of this case.

Tourniquet‐associated complications present a diverse clinical spectrum, with tourniquet pain representing the most prevalent early adverse effect. Studies indicate that up to 66% of patients may experience pain or discomfort at the cuff site or in the distal limb approximately 30–60 min after tourniquet inflation [1, 6]. However, spinal anesthesia induces a distinct physiological state by initially blocking somatic sensory afferents (Aδ and C fibers) from spinal segments below L3 [7]. After intrathecal administration of an adequate dose of local anesthetic (LA), conduction in both A‐ and C‐fibers is blocked. However, as the concentration of LA in the CSF decreases, the C‐fibers start conducting impulses before the A‐fibers, resulting in a dull tourniquet pain in the presence of an otherwise adequate surgical anesthesia [8]. Therefore, conventional nociceptive signals originating from ischemic muscle distal to the tourniquet cannot be fully suppressed and may ascend via the partially recovered C‐fiber pathways to reach conscious perception [1, 9]. This renders the classic presentation of tourniquet pain neither accurately perceived nor precisely localized; instead, it may manifest as a vague, poorly defined discomfort or “referred pain” within nonblocked neural segments.

Referred pain is defined as pain perceived and localized to a superficial or cutaneous region distant from the actual site of pathology or stimulation within visceral organs or deep somatic tissues. This common clinical phenomenon is primarily explained by the convergence‐projection theory in neuroanatomy [10]. Reports indicate that spinal referred pain manifests in the lumbar region at a notable incidence ranging from 17% to 84% [11]. In the present case, profound ischemia, acidosis, and cellular injury within the deep tissues (e.g., muscle) of the patient’s right thigh release a cascade of mediators, including potassium and hydrogen ions, adenosine, bradykinin, and inflammatory cytokines such as substance P and IL‐1β [12]. These substances not only stimulate local nociceptive terminals but may also activate sympathetic afferent fibers accompanying vascular structures or visceral‐type afferents sensitive to chemical stimuli. Evidence suggests that regional anesthesia may incompletely block such fiber types. The observed tourniquet‐induced hypertension in this patient, evidenced by a progressive rise in blood pressure, further supports the involvement of these alternative signaling pathways. Sympathetic nervous system activity, which is crucial for maintaining vascular tone and arterial pressure, is highly dependent on enhanced sympathetic outflow [13]. Thus, the hypertensive response aligns with the engagement of sympathetic‐mediated afferent signaling, reinforcing the plausibility of the proposed referred pain mechanism in this unique clinical scenario.

These aberrant visceral afferent signals from the deep tissues of the lower limb ascend to the spinal cord via sympathetic or visceral fiber pathways that remain incompletely blocked. They likely synapse within the dorsal horn of spinal segments T10‐L2, a region that receives both visceral input from the lower limb and somatic sensory input from the skin of the lower back. According to the classic convergence‐projection theory [10, 14], when afferent signals from visceral and somatic sources converge onto the same spinal neuron, the cerebral cortex cannot distinguish their origin and typically “projects” the pain to the more familiar cutaneous region. Consequently, the brain misinterprets the tourniquet‐induced ischemic signal as originating from the skin and muscles of the lower back, leading the patient to experience well‐localized, severe lumbar pain. Upon tourniquet release, the ischemic stimulus ceases, nociceptive signaling terminates, and symptoms resolve abruptly.

This study reveals a significant knowledge gap: current surgical, anesthesiology literature, textbooks, and guidelines scarcely mention that tourniquet pain under regional anesthesia may present as intense, isolated referred pain remote from the ischemic site. Our case demonstrates that the mode of anesthesia can fundamentally reshape the clinical manifestation of this complication. This omission directly contributes to the risk of clinical misdiagnosis. Therefore, when an anesthesiologist encounters sudden‐onset truncal pain (e.g., lumbar and abdominal) in a patient under regional anesthesia, after excluding common causes, it is imperative to actively assess tourniquet duration and consider it as a potential culprit. A simple “tourniquet‐release test” may serve as a diagnostic intervention. Concurrently, surgeons should strictly adhere to tourniquet guidelines, limiting single‐application duration (generally recommended ≤ 1.5 h) and employing pneumatic tourniquets capable of precise pressure control [15]. Close communication between the surgical and anesthesia teams regarding tourniquet status is essential.

## 5. Conclusion

This case provides the first documented evidence that tourniquet‐induced ischemia under spinal anesthesia can manifest as acute, severe, and atypical lower back pain, with immediate resolution upon tourniquet deflation. We propose that the underlying mechanism involves ischemic signals ascending via visceral or sympathetic pathways incompletely blocked by the spinal anesthetic, leading to their erroneous spatial referral as lumbar pain at the spinal cord level. This finding refines and expands the clinical phenotype of “tourniquet‐associated complications,” underscoring how their presentation can be fundamentally altered under specific anesthetic conditions. Enhancing awareness of this atypical manifestation holds direct clinical value for enabling early and accurate diagnosis, preventing unnecessary interventions, and ultimately improving patient safety.

## 6. Limitations and Future Perspectives

First, the use of a nonpneumatic elastic tourniquet, rather than a modern pneumatic device, introduced substantial limitations in pressure regulation and cuff‐width selection. Of note, nonpneumatic elastic tourniquets are associated with a markedly higher incidence of tourniquet‐related pain and tissue irritation compared with standard pneumatic tourniquets. This scenario reflects common practical constraints encountered in resource‐limited clinical settings, including certain regions in Africa. Second, the lack of standardized safety time limits for tourniquet application, together with the absence of an automatic deflation mechanism, led to repeated prolongation of tourniquet use at the surgeon’s discretion. Such practice not only complicated anesthetic management but also heightened the risk of ischemia‐related adverse events. Third, when refractory lower back pain arose after prolonged tourniquet inflation beyond the recommended safe duration, the initial intervention consisted of supplemental analgesic administration rather than prompt tourniquet release. This strategy increased cumulative opioid consumption and deviated from the current principle of opioid‐sparing anesthetic practice.

Future research should conduct prospective controlled studies to better define the incidence of this underrecognized clinical phenomenon and to explore its associations with anesthetic type and concentration, tourniquet pressure, application duration, tourniquet type, and individual patient characteristics. Further neurophysiological assessments or functional imaging investigations may help validate the hypothesized mechanism of referred pain underlying this atypical presentation.

## Funding

No funding was received for this manuscript.

## Consent

Written informed consent was obtained from the patient for publication of this case report and any accompanying images. A copy of the written consent is available for review by the Editor of this journal.

## Conflicts of Interest

The authors declare no conflicts of interest.

## Supporting Information

Additional supporting information can be found online in the Supporting Information section.

## Supporting information


**Supporting Information** Supporting Information 1 presents the complete Schatzker tibial plateau fracture classification system, which provides standardized fracture grading criteria referenced in the Case History section (Supporting Information 1 [16]). This supporting information supports the clinical typing judgment of the patient’s fracture in this case report.

## Data Availability

The data supporting the findings of this study are available from the corresponding author upon reasonable request.

## References

[1] Kamath K. , Kamath S. U. , and Tejaswi P. , Incidence and Factors Influencing Tourniq-uet Pain, Chinese Journal of Traumatology. (September 2021) 24, no. 5, 291–294, 10.1016/j.cjtee.2021.05.002. 34281783 PMC8563858

[2] Albaker A. B. , Almogbil I. , Alkheraiji A. F. et al., Tourniquet Practice Among Orthopaedic Surgeons in Saudi Arabia, Cureus. (September 2023) 15, no. 9, 10.7759/cureus.45828. PMC10591230 37876395

[3] Fu Q. , Han M. , Mu Y. et al., Does the Pain Sensitivity Questionnaire Correlate With Tourniquet Pain in Patients Undergoing Ankle Surgery?, Frontiers in Surgery. (February 2023) 10, 10.3389/fsurg.2023.1102319. PMC10009183 36923376

[4] Chang J. , Bhandari L. , Messana J. , Alkabbaa S. , Hamidian Jahromi A. , and Konofaos P. , Management of Tourniquet-Related Nerve Injury (TRNI): A Systematic Review, Cureus. (August 2022) 14, no. 8, 10.7759/cureus.27685. PMC9440764 36072167

[5] Sharma J. P. and Salhotra R. , Tourniquets in Orthopedic Surgery, Indian Journal of Orthopaedics. (2012) 46, no. 4, 377–383, 10.4103/0019-5413.98824. 22912509 PMC3421924

[6] Swan K. G.Jr., Wright D. S. , Barbagiovanni S. S. , Swan B. C. , and Swan K. G. , Tourniquets Revisited, The Journal of Trauma. (March 2009) 66, no. 3, 672–675, 10.1097/TA.0b013e3181986959. 19276736

[7] Gropper M. A. , Eriksson L. I. , Fleisher L. A. et al., Miller’s Anesthesia, Chapter 41, Spinal, Epidural, and Caudal Anesthesia. (2024) 10th edition, Elsevier, Philadelphia, Pennsylvania, 1482–1486.

[8] Gissen A. J. , Covino B. G. , and Gregus J. , Differential Sensitivities of Mammalian Nerve Fibers to Local Anesthetic Agents, Anesthesiology. (December 1980) 53, no. 6, 467–474, 10.1097/00000542-198012000-00006. 7457962

[9] Guo M. and Sun J. , Research Progress on the Mechanism, Prevention, and Treatment of Tourniquet Pain, Chinese Journal of General Practice and Clinical Education. (2024) 22, no. 2, 161–163, 10.13558/j.cnki.issn1672-3686.2024.002.017.

[10] Arendt-Nielsen L. and Aalborg U. , Lab for Experimental Pain Research, Ctr for Sensory-Motor Interaction, Aalborg, Denmark;Svensson, Peter.Referred Muscle Pain: Basic and Clinical Findings, The Clinical Journal of Pain. (2001) 17, no. 1, 11–19, 10.1097/00002508-200103000-00003. 11289083

[11] Derby R. , Lee S. H. , Seo K. S. , Kazala K. , Kim B. J. , and Kim M.J. , Efficacy of IDET for Relief of Leg Pain Associated With Discogenic Low Back Pain, Pain Practice. (2004) 4, no. 4, 281–285, 10.1111/j.1533-2500.2004.04401.x. 17173608

[12] Murata I. , Nozaki R. , Ooi K. et al., Nitrite Reduces Ischemia/Reperfusion-Induced Muscle Damage and Improves Survival Rates in Rat Crush Injury Model(Article), Journal of Trauma and Acute Care Surgery. (2012) 72, no. 6, 1548–1554, 10.1097/TA.0b013e31824a76b5. 22695420

[13] Kim E. , Cho M. R. , and Byun S. H. , Sympathetic Predominance Before Tourniquet Deflation is Associated With a Reduction in Arterial Blood Pressure After Tourniquet Deflation During Total Knee Arthroplasty, Physiological Research. (2021) 70, no. 3, 401–412, 10.33549/physiolres.934639. 33982581 PMC8820555

[14] Arendt-Nielsen L. , Headache: Muscle Tension, Trigger Points and Referred Pain, International Journal of Clinical Practice. (2015) 69, no. 182, 8–12, 10.1111/ijcp.12651. 25907017

[15] Kneisley M. , Guidelines in Practice: Pneumatic Tourniquet Safety, AORN Journal. (November 2025) 122, no. 5, 309–317, 10.1002/aorn.14425. 41163527

[16] Schatzker J. , McBroom R. , and Bruce D. , The Tibial Plateau Fracture. The Toronto Experience 1968–1975, Clinical Orthopaedics and Related Research. (January 1979) 138, 94–104, https://pubmed.ncbi.nlm.nih.gov/445923/. 445923

