# Supplementary material for: Tourniquet‐Induced Atypical Lower Back Pain Under Spinal Anesthesia: A Case Report and Mechanistic Exploration of Referred Pain
Source: Case Rep Med. 2026 Jun 29;2026:5531848. doi: 10.1155/carm/5531848 (PMC13311721; doi:10.1155/carm/5531848)
Supplement: Supplementary file 1 — Supporting Information Supporting Information 1 presents the complete Schatzker tibial plateau fracture classification system, which provides standardized fracture grading criteria referenced in the Case History section (Supporting Information 1 [16]). This supporting information supports the clinical typing judgment of the patient’s fracture in this case report. [file CARM-2026-5531848-s001.docx]

Supplementary Material 1:Classification criteria for tibial plateau fractures


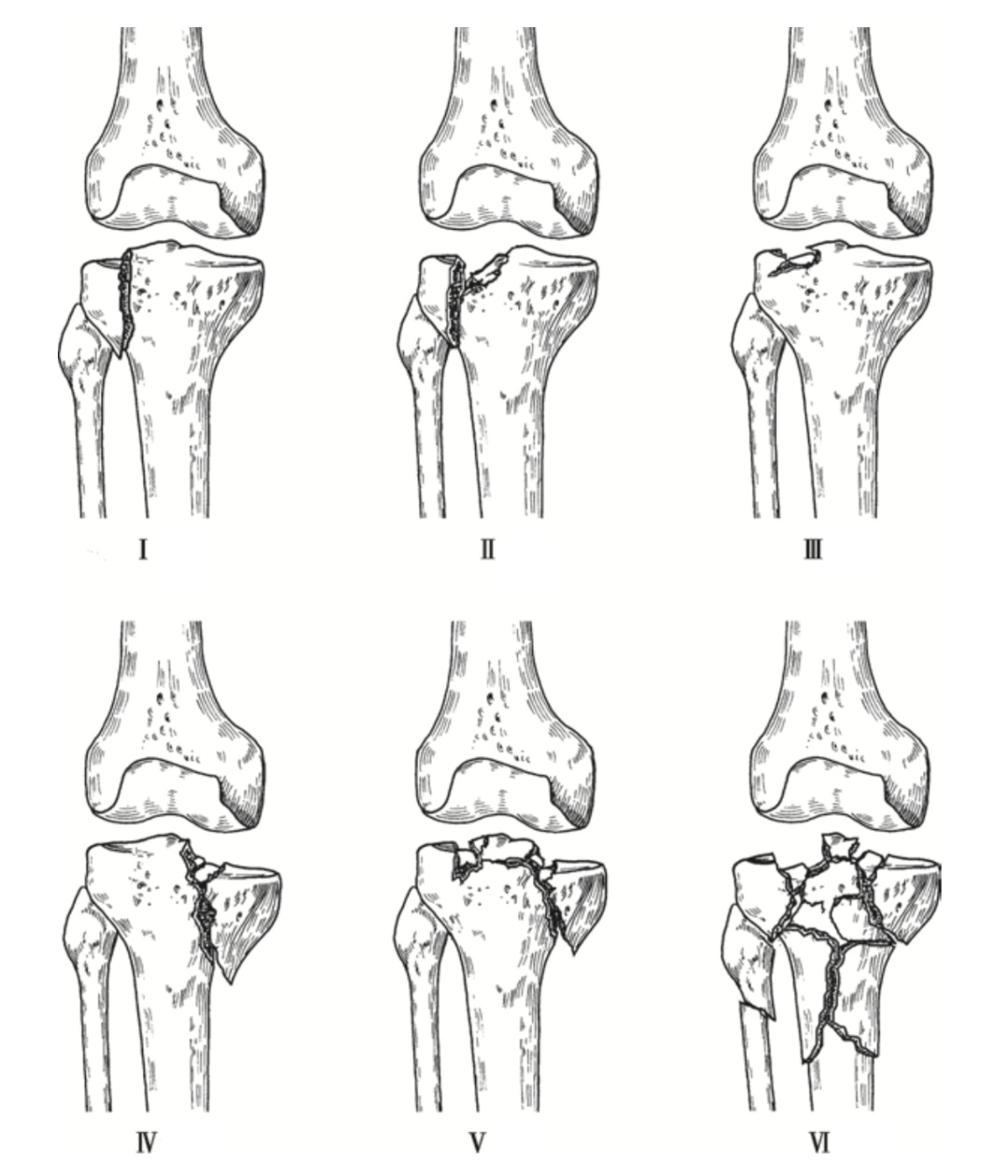


Figure of the plateau fracture classification

Type I: Isolated split fracture of the lateral tibial plateau, without associated articular surface depression.

Type II: Split-depression fracture of the lateral tibial plateau, consisting of a lateral split combined with articular surface subsidence.

Type III: Pure depression fracture confined to the lateral tibial plateau, with no metaphyseal split component.

Type IV: Fracture of the medial tibial plateau, presenting as either an isolated split fracture or a combined split-depression injury.

Type V: Bicondylar tibial plateau fracture, with variable degrees of articular surface depression and condylar displacement on both medial and lateral sides.

Type VI: Tibial plateau articular fracture combined with metaphyseal-diaphyseal dissociation (separation of the tibial metaphysis from the tibial shaft).
